# Histones and Their Modifications in Ovarian Cancer – Drivers of Disease and Therapeutic Targets

**DOI:** 10.3389/fonc.2014.00144

**Published:** 2014-06-12

**Authors:** Deborah J. Marsh, Jaynish S. Shah, Alexander J. Cole

**Affiliations:** ^1^Hormones and Cancer Group, Kolling Institute of Medical Research, Royal North Shore Hospital, The University of Sydney, Sydney, NSW, Australia

**Keywords:** histone, ovarian cancer, splicing, lncRNA, polycomb repressive complex, histone deacetylase inhibitors, deubiquitinases, histone methyltransferases

## Abstract

Epithelial ovarian cancer has the highest mortality of the gynecological malignancies. High grade serous epithelial ovarian cancer (SEOC) is the most common subtype, with the majority of women presenting with advanced disease where 5-year survival is around 25%. Platinum-based chemotherapy in combination with paclitaxel remains the most effective treatment despite platinum therapies being introduced almost 40 years ago. Advances in molecular medicine are underpinning new strategies for the treatment of cancer. Major advances have been made by international initiatives to sequence cancer genomes. For SEOC, with the exception of *TP53* that is mutated in virtually 100% of these tumors, there is no other gene mutated at high frequency. There is extensive copy number variation, as well as changes in methylation patterns that will influence gene expression. To date, the role of histones and their post-translational modifications in ovarian cancer is a relatively understudied field. Post-translational histone modifications play major roles in gene expression as they direct the configuration of chromatin and so access by transcription factors. Histone modifications include methylation, acetylation, and monoubiquitination, with involvement of enzymes including histone methyltransferases, histone acetyltransferases/deacetylases, and ubiquitin ligases/deubiquitinases, respectively. Complexes such as the Polycomb repressive complex also play roles in the control of histone modifications and more recently roles for long non-coding RNA and microRNAs are emerging. Epigenomic-based therapies targeting histone modifications are being developed and offer new approaches for the treatment of ovarian cancer. Here, we discuss histone modifications and their aberrant regulation in malignancy and specifically in ovarian cancer. We review current and upcoming histone-based therapies that have the potential to inform and improve treatment strategies for women with ovarian cancer.

## Introduction

Epithelial ovarian cancer has the highest mortality of all of the gynecological malignancies, with high grade serous epithelial ovarian cancer (SEOC) the most common subtype. Due to general or non-descript symptoms of early stage disease, the majority of women initially present with advanced malignancy (Stage III or IV) where 5-year survival can be as low as 25% (1, 2). Standard of care is surgical debulking followed by combinations of platin-based drugs such as carboplatin with paclitaxel [reviewed in Ref. (3)]. Cisplatin was first approved by the Food and Drug Administration (FDA) for the treatment of ovarian cancer in 1978 (4), while paclitaxel was approved in 1992 (5). Some evidence exists to support the success of neoadjuvant chemotherapy in women who present with advanced, unresectable primary ovarian cancer, followed by interval debulking; however, data also exist suggesting there is little or no benefit to this approach (6, 7). Most women respond to standard of care chemotherapeutic drugs initially; but the majority relapse within 2 years, ultimately developing broad chemoresistance (8, 9).

Additional factors complicating the success of current treatment strategies for SEOC is lack of a clear understanding of the true site and cells of origin of this malignancy, with evidence mounting that SEOC may in fact arise in the secretory fimbrial cells of the fallopian tube (10, 11). Molecular heterogeneity of ovarian cancer also poses challenges, with distinct molecular subtypes based on gene expression identified within identical histopathological groupings such as SEOC (12). Knowledge of post-translational histone modifications associated with cancer, including ovarian cancer, is emerging. This review discusses histones and their post-translational modifications (PTMs) as key regulators of gene expression and DNA repair with relevance for the treatment of ovarian cancer.

## Genetics and Genomics of SEOC, Informing New Therapies

While advances in genetics have not fully addressed the challenges of treating ovarian cancer, elucidation of the mutational SEOC landscape is informing the development of therapies targeting DNA damage signaling pathways. Extensive international efforts channeled into sequencing a large cohort of sporadic SEOC through The Cancer Genome Atlas (TCGA) project has been revealing. With the exception of *TP53* that is mutated in almost 100% of these cancers, there is a relatively low frequency of mutations (approximately 2–6%) in genes including *BRCA1, BRCA2, CSMD3, NF1, CDK12, FAT3, GABRA6*, and *RB1*, that might otherwise have been more directive for therapeutic targeting (13). Determining the role of multiple gene mutations in relation to the activation of cancer-associated signaling pathways for individual tumors will however be of value for guiding targeted therapies. The development of strategies to target mutant p53 proteins will clearly have major relevance to SEOC (14). Furthermore, large cohort studies of primary SEOC and SEOC cell line models have revealed extensive copy number variations that would function as a major driver of aberrant gene expression (13, 15).

Creating great excitement in this field is the introduction of a new class of drugs known as [poly (ADP-ribose) polymerase, PARP] inhibitors, including drugs such as Olaparib^®^ (AZD2281, AstraZeneca), Rucaparib^®^ (AG 014699, Clovis), and Veliparib^®^ (ABT-888, Abbot) (16, 17). PARP1 is important in the cellular response to DNA damage, binding to single and double-strand breaks where it mediates recruitment of factors activated in the DNA damage response such as the serine/threonine protein kinase Ataxia telangiectasia mutated (ATM) (18). In cells lacking functional homologous recombination pathways, e.g., with mutation, silencing, or other functional dysregulation of proteins involved in DNA repair such as BRCA1 and BRCA2, PARP1 inhibition leads to persistent double-strand breaks and cell death. This is particularly relevant to SEOC where aberrations in DNA damage pathways are well recognized as major driver of these tumors. The frequency of germline *BRCA1* and *BRCA2* mutation in familial ovarian cancer is around 17% (19, 20). While encouraging, not all women with SEOC respond to PARP1 inhibition, and some that do will develop resistance. Key molecular drivers of PARP1 sensitivity and resistance are beginning to be elucidated (21–23) and trials of PARP1 inhibitors have shown promise (24). It is interesting to speculate that manipulation of factors involved in chromatin accessibility may have the potential to increase the success of PARP1 inhibitors that are undoubtedly an exciting new therapeutic option for SEOC.

## Epigenomics and SEOC, Unlocking New Opportunities for Therapy

Aberrant DNA methylation and microRNA (miRNA) expression have also been identified in SEOC (25, 26). DNA methylation refers to the addition of a methyl group to the cytosine-5 position of a CpG dinucleotide that is controlled by DNA methyltransferases. There are well described cases of gene regulation in ovarian cancer relying on hyper- or hypomethylation, including down-regulation of both *BRCA1* and the *PTEN* tumor suppressors by promoter hypermethylation (27, 28). Of note, the cell surface marker CD133 that is part of a panel used to define ovarian cancer-initiating cells has been shown to be regulated by both histone modification and promoter methylation (29). Other cancer-associated genes with increased expression in ovarian cancer due to promoter hypomethylation include *TUBB3* and *HOXA10* (30, 31). Epigenetic silencing of genes has been linked to the development of platin-based resistance in ovarian cancer, including DNA hypermethylation at CpG sites of *MLH1, ARMCX2, COL1A1, MDK*, and *MEST* gene promoters (26, 32). Treatment of cisplatin resistant human ovarian cancer cell line xenografts with the demethylating agent 5-aza-2′-deoxycytidine resensitized tumors to platin-based therapy, likely through re-expression of MLH1 associated with a decrease in *MLH1* promoter hypermethylation (33). While unlikely to be efficacious as monotherapy, the value of demethylating agents for the treatment of ovarian cancer may be in combinatorial treatments with more conventionally used DNA damaging agents such as the platin-drugs or other epigenomic-based therapies. Interactions between histone modifications and DNA methylation that together influence gene expression have been reported (34). A number of reviews addressing the topic of DNA methylation in ovarian cancer, including discussion of clinical trials of demethylating agents, are available (25, 35, 36).

Elucidation of the role of post-translational histone modifications and parallel development of therapeutic strategies targeting them is gaining momentum in many tumor streams; however, this area of epigenomics is to date relatively understudied in ovarian cancer, although examples of this form of gene regulation are emerging. Targeting histone modifications has the potential to be of particular relevance to the treatment of SEOC given that these strategies embrace a whole genome approach, and so have the potential to overcome issues created by focusing on infrequently mutated genes. Furthermore, many histone modifications have been implicated in the DNA damage response given their fine control of chromatin configuration that determines access by transcription factors and DNA repair proteins (37). SEOC is undoubtedly a tumor driven by aberrant DNA damage signaling, therefore the potential exists to improve the way this pathway is targeted with current therapies by a greater understanding of the chromatin landscape. It has recently been stated that we stand at the “*tipping point*” for epigenetic based therapies for the treatment of cancer (38). The strategies being developed have large potential for the treatment of ovarian cancer.

## Modifying Core Histones

### Post-translational histone modifications

Histones are small basic proteins of around 14 kDa that contain a high percentage of positively charged amino acids (39). They are the most abundant proteins bound to DNA in eukaryotic cells and predominantly function to regulate gene expression and DNA packaging around nucleosomes, the functional units of chromatin. Nucleosomes are comprised of a histone octamer with two copies each of the core histone proteins H2A, H2B, H3, and H4 wrapped around by approximately 147 bp of DNA (39). Within this structure, H3:H4 exists as a tetramer and there are two H2A:H2B dimers (40). The histone linker H1 binds nucleosomes together thereby participating in a higher order compaction of chromatin (41). NH_2_-terminal histone tails protrude from the core octamer structure, with residues located in these tails subject to a large number of dynamic and reversible PTMs that include, but are not limited to, methylation, acetylation, phosphorylation, ubiquitination, and SUMOylation (42).

Post-translational modifications of core histone proteins regulate gene transcription, replication, and DNA repair processes by influencing chromatin configuration and providing important platforms or docking sites for the recruitment of proteins and enzyme complexes such as methyltransferases and acetylases required for chromatin modeling. New terminology has recently entered this field describing chromatin “writers” that lay down histone modifications, chromatin “erasers” that remove them, and chromatin “readers” that are involved in interpretation of signals that may influence subsequent changes (40). Histone H3 lysine 4 di- and tri-methylation (H3K4me2 and H3K4me3), as well as histone H3 lysine 79 methylation (H3K79me), histone H3 lysine 36 (H3K36me), histone acetylation and monoubiquitination of histone H2B at lysine 120 (H2Bub1) have been linked to “open” chromatin and active transcription. Other modifications, including methylation of histone H3 lysine 9 (H3K9me), histone H3 lysine 27 (H3K27me), and histone H4 lysine 20 (H4K20me) are associated with “closed” chromatin and transcriptional repression (42, 43). Suppression of H3K27me3 in cell lines overexpressing the dominant negative mutant H3-K27R led to re-expression of the RASSF1 tumor suppressor and resensitization of ovarian cancer cells to cisplatin, likely due to a more relaxed and open chromatin configuration (44). Methylation is controlled in a reversible fashion by methyltransferases and demethyltransferases, often associating in complexes, whilst monoubiquitination is dynamically controlled by ubiquitin ligases such as the RING finger proteins RNF20 and RNF40 (45, 46) and deubiquitinases (DUBs), again often in complex structures.

Histone acetylation is generally associated with an open chromatin structure that facilitates transcription, controlled in a dynamic fashion by histone acetyltransferases (HATs) and histone deacetyltransferases (HDACs) (47). Acetylation acts to neutralize the positive charge of lysine residues located on histone tails, resulting in disruption of nucleosomal structure and promoting unfolding of local DNA making it more accessible by transcription machinery. HDACs remove acetyl residues and consequently are associated with gene repression. In many cancers including ovarian, aberrant HDAC pathways are believed to promote cancer growth and metastasis (48–50). Histone tail residues can provide platforms for multiple enzyme writers, such as lysine 120 of histone H2B that in addition to being monoubiquitinated, can also be acetylated (H2BK120ac). It is thought in this case that H2BK120ac precedes H2Bub1 in a temporal fashion, suggesting that it may be an early mark of poised or active chromatin functioning as a dual switch to keep nucleosomes “hot” for rounds of induction and transcriptional elongation (51).

Histone deacetyltransferases that are aberrantly expressed in cancer include sirtuins of which there are seven family members. Sirtuins are mammalian homologs of the yeast silent information regulator (Sir2), that as well as functioning as HDACs can act as deacetylases for non-histone proteins such as p53 (52). SIRT1 is a nicotinamide adenine dinucleotide (NAD^+^) – dependent lysine deacetylase and a class III HDAC. SIRT1 expression was reported to be higher in malignant EOC compared to benign, and expression was seen more commonly in SEOC relative to mucinous tumors (53). This same study reported higher levels of SIRT1 in a subset of malignant SEOC that correlated with increased overall survival. Of note, BRCA1-associated breast cancers have been reported to have lower levels of SIRT1 relative to BRCA1 wild-type (54). To date, levels of SIRT1 in BRCA1-associated EOCs have not been assessed. SIRT1 is also associated with acquired drug resistance, influencing the tumor microenvironment, functioning in DNA repair and promoting cancer stem cell survival (55). For all these reasons, SIRT1 is being considered as a possible target to overcome drug resistance seen in many malignancies and may have relevance to future treatment strategies for SEOC.

There is emerging evidence to support the theory that sub-populations of cells exist in SEOC that are of a stem cell-like nature, demonstrate resistance to chemotherapy and are responsible for the development of chemoresistance in women with ovarian cancer (56–58). This is supported by a recent study demonstrating that the bivalent chromatin mark seen in embryonic stem cells and required for silencing of developmental genes, H3K27me3/H3K4me3, is found in SEOC at the transcription start sites of silenced genes (59). H3K27 methylation is written by the methyltransferase Enhancer of Zeste Homolog 2 (EZH2) that forms the catalytic unit of Polycomb repressive complex 2 (PRC2). EZH2 is overexpressed in SEOC, as well as in cancer-associated stromal cells (60). H3K27me has also been associated with chemoresistance in ovarian cancer (59). Histone PTMs play a major role in maintenance of an undifferentiated stem cell phenotype (61, 62).

Studies of histone PTMs in primary tumors support discoveries in cancer cell line models of complex histone modifications and furthermore, have been shown to correlate with tumor stage and prognosis. Loss of global H3K27me3 has been shown in ovarian, as well as breast and pancreatic cancers, correlating with shorter overall survival (63). Loss of global levels of H2Bub1 has been reported in advanced breast tumors, as well as colon, lung, parathyroid, and ovarian cancers (64–67).

### Cross-talk between histone modifications

**Figure 1:**
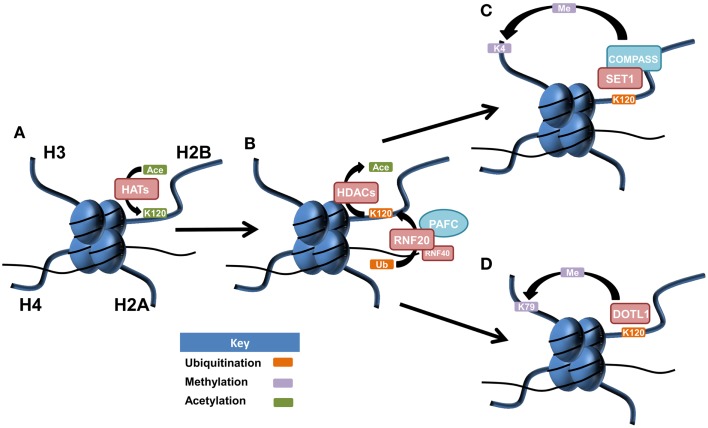
**Postulated patterns of histone cross-talk in malignancy**. **(A)** Lysine 120 of histone H2B is acetylated by histone acetyltransferases (HATs), acting as a precursor for histone H2B monoubiquitination at the same amino acid residue. **(B)** Lysine 120 of histone H2B becomes deacetylated *via* histone deacetylases (HDACs), allowing for the E3 ubiquitin ligase complex of RNF20/RN40, in association with the PAF1 transcriptional regulatory complex (PAFC) to facilitate monoubiquitination of lysine 120 (H2Bub1). **(C)** SET1 is recruited to the site of H2Bub1 where it interacts with COMPASS (complex of proteins associated with Set1) to facilitate the active mark of methylated histone 3 at lysine 4 (H3K4me). **(D)** H2Bub1 can recruit and activate the DOT1L methyltransferase, responsible for the active chromatin mark of methylated histone H3 at lysine 79 (H3K79me).

Histone cross-talk is defined as the influence that one or more post-translationally modified histones have on the writing, erasing, and reading of other histone PTMs. The language of histones is both complex and wide spread, influencing processes involved in development, stem cell differentiation, transcription, replication, and DNA repair with a major role in the regulation of gene expression (68, 69). Examples of histone cross-talk include the recruitment of the methyltransferase complex COMPASS (complex of proteins associated with Set1) by H2Bub1 that is involved in the methylation of lysine 4 on histone H3 (70–73) (Figure 1). This cross-talk also has implications for DNA damage signaling as methylated H3K4 recruits the DNA damage-associated chromatin remodeling factor SNF2H leading to recruitment of DNA repair proteins RAD51 and BRCA1 (74, 75). The DOT1L methyltransferase has been shown to methylate H3K79 after its expression was first stimulated by increased H2Bub1 (76). Complex patterns of cross-talk and their influence on gene expression and cellular processes are only just beginning to be elucidated. This will be an area of considerable focus in the future given the importance of understanding how therapies targeting one histone modification may in fact be influencing another.

### Differential histone splicing

The study of histone splice variants in ovarian cancer is still in its infancy, with few studies published to date, although evidence suggests roles in tumor progression (77). Slight structural changes to the core histone octamer as the result of incorporation of differentially spliced histones can alter the overall structure of the nucleosome, changing the way in which DNA wraps around it and influencing nucleosome dynamics (78). These non-canonical variants can influence the function of chromatin domains and lead to differences in nucleosome stability causing aberrant transcription and DNA repair (79). Roles for histone splicing are just beginning to be elucidated in ovarian cancer. A link between alternative histone splicing of a group of H2A-type histone variants, referred to as macroH2As (macroH2A1.1, macroH2A1.2, and macroH2A2), and proliferation has been reported in a number of cancers, including ovarian (80). The RNA binding protein QKI (Quaking) was shown to regulate alternative pre-mRNA splicing of macroH2A1. Interestingly, macroH2A1.1-mediated suppression of proliferation occurs, at least in part, through the reduction of PARP1 protein levels. Given the interest in PARP1 inhibition for therapy, this area requires further attention.

Another histone variant identified to be down-regulated in ovarian cancer is histone variant H2A.Z, loss of which resulted in tumor progression (81). H2A.Z is a conserved variant of histone H2A, and has recently been shown to regulate a variety of targets including the glucocorticoid receptor (82), estrogen receptor (83), and p53 (84). The ovarian cancer cell lines A2780 and OVCAR3 were shown to have lost H2A.Z from regulatory regions of the urokinase receptor (u-PAR) leading to activation of this receptor and suggesting a mechanism for upregulation of u-PAR that is seen in a number of different malignancies (81, 85, 86). Furthermore, expression of linker histone H1 splice variants has been shown to discriminate ovarian adenocarcinomas from adenomas, suggesting their value as potential epigenetic biomarkers of ovarian cancer (87).

## Role of Non-Coding RNAs in the Regulation of Histones

The surprising finding that at least 80% of the human genome is transcribed has boosted an interest in understanding the role of non-coding RNAs (lncRNAs) in biological processes and diseases. The number of protein coding genes has remained relatively stable at approximately 21,000 during the last decade; however, the number of lncRNAs has grown to 9000 small lncRNAs (<200 nt) and 10,000–32,000 long lncRNAs (>200 nt) (88).

The role of miRNAs, a subset of small lncRNAs, in regulation of post-transcriptional gene silencing has been well established; however, our understanding of their effects on biological networks is still far from complete (88). Global deregulation of miRNAs has been implicated in ovarian cancer, and miRNAs have been found to target DICER, a key enzyme in miRNA processing, in breast cancer (13, 89–92). Of note, DICER levels do not appear to be altered in ovarian cancer (91). DNA methyltransferases such as DNMT1 and DNMT3B, histone deacetylases such as HDAC2 and HDAC4, and HATs such as KAT2B and KAT6A themselves are predicted to be targeted by dysregulated miRNAs in ovarian cancer (miR-100, 140, 145, 21, 26a, and 93) according to miRTarBase, a database of experimentally validated miRNA targets, influencing the epigenome (93).

### HOTAIR, EZH2, and the polycomb repressive complex

**Figure 2:**
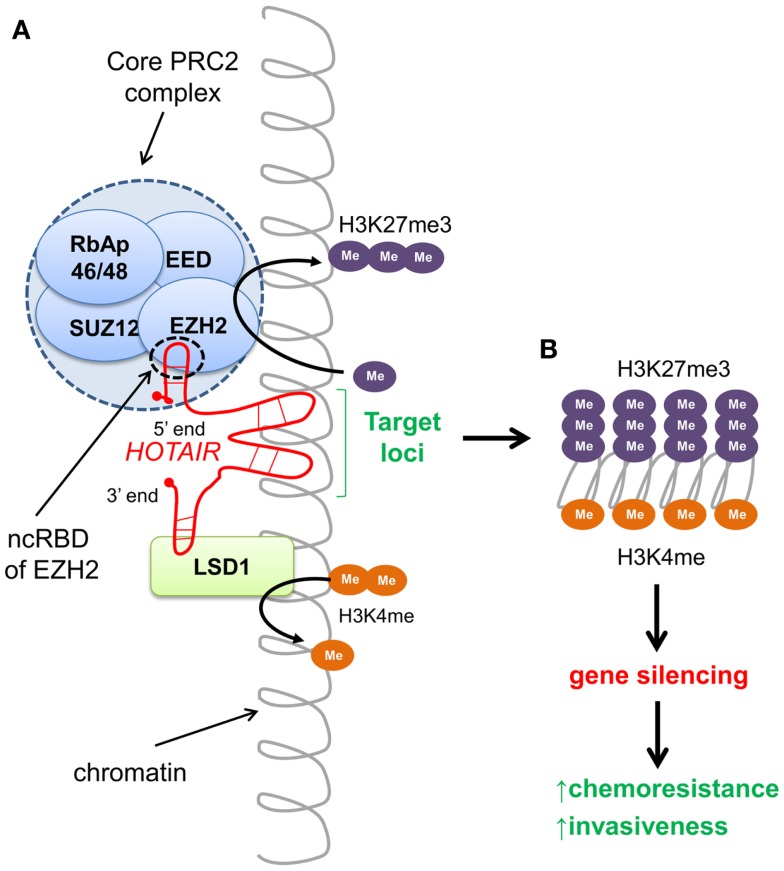
***HOTAIR*-directed epigenetic reprograming of the cancer genome**. **(A)** The long intergenic non-coding (linc) RNA *HOTAIR* recognizes specific DNA sequences and targets chromatin-modifying complexes PRC2 and LSD1 to silence gene loci. The 5′ end of *HOTAIR* tethers the PRC2 complex to the target by binding to the non-coding RNA binding domain (ncRBD) of the HMTase EZH2, catalyzing tri-methylation of H3K27. The 3′ end of *HOTAIR* facilitates demethylation of H3K4me2 by the lysine-specific demethylase LSD1. Both H3K27me3 and lack of methylation at H3K4 are repressive chromatin marks associated with gene silencing. **(B)** Expression of *HOTAIR* results in silencing of >40-kb region spanning *HOXD8–11* of the *HOXD* locus. Aberrant *HOTAIR* expression in multiple cancers has been shown to promote invasiveness.

Non-coding RNAs are versatile, with roles ranging from chromatin structure modification, X chromosome inactivation, scaffold function, miRNA decoys, nuclear import and export, RNA splicing, and the regulation of gene expression (94–96). Recently, approximately 20% of long intergenic lincRNAs, a subset of lncRNAs, have been found to be associated with PRC2 that functions as a chromatin-modifying complex (97). *HOTAIR* (*HOX* transcript antisense intergenic RNA), an approximately 2.1 kb lincRNA transcribed from the *HOXC* locus, is known to alter chromatin configuration and promote cancer. In healthy cells, *HOTAIR* functions to epigenetically silence approximately 40 kb spanning *HOXD8–HOXD11* of the *HOXD* region on chromosome 2 (98, 99). By working as a scaffold, *HOTAIR* tethers and directs the PRC2 containing the H3K27 methylase EZH2, and the lysine-specific demethylase 1 (LSD1) to silence targets by catalyzing H3K27me3 and demethylating H3K4me2, depicted in Figure 2 (100).

Over-expression of *HOTAIR* promotes metastasis of breast, pancreatic, endometrial, colorectal, and other cancers (99, 101–104). Conversely, silencing of *HOTAIR* impaired migration and invasion of EOC cells *in vitro*, as well as inhibited tumor spread in a mouse model of intraperitoneal metastasis, likely *via* metalloproteinases (MMP3 and matrix metalloproteinase-9, MMP9) and epithelial-to-mesenchymal pathways (105). *HOTAIR* levels were reported to be elevated in ovarian cancer relative to normal ovary, and its expression was inversely correlated with the degree of differentiation (101, 105). Elevated levels of *HOTAIR* correlated with worse overall and disease free survival in women with EOC and were also correlated with the presence of lymph node metastasis (105). Furthermore, *HOTAIR* is expressed at a fivefold higher level in cisplatin resistant A2780cisR cells compared to parental A2780 cells, and its down-regulation restored cisplatin sensitivity (106). Levels of *HOTAIR* have been reported to be fourfold higher in colon and breast cancer stem cell-like cells (CD133^+^/CD44^+^) compared to non-stem cell-like cells (CD133^−^/CD44^−^), and its down-regulation reduced the number and size of colonies assessed by anchorage-independent growth (107). *HOTAIR* was also shown to induce epithelial-to-mesenchymal transition (EMT) following TGF-β1 treatment in colon and breast cancer cell lines (107).

Polycomb-group (PcG) proteins are involved in maintaining the repression of genes in specific cells and subsequent cells originating from them. These proteins are essential in lineage commitment where they and their antagonists, Trithorax proteins, selectively express and repress a subset of *HOX* genes required to specify a particular cell type. Tumor suppressors including p16^Ink4a^, p19^Arf^, and p15^Ink4b^ are epigenetically silenced due to abrogation of PcG proteins in cancer (108). Most PcG proteins form multimeric complexes of either Polycomb repressive complex 1 (PRC1) or 2 (PRC2). Mammalian PRC2 complex comprises four core PcG proteins: EED, SUZ12, EZH1/2, and RbAp46/48, with many other proteins interacting with this core complex. EZH1 and EZH2 are histone methyltransferases (HMTases) and form part of the PRC2 complex that initiates gene silencing. As HMTases, gene silencing is enabled as both EZH1 and EZH2 contain SET domains required to catalyze di- or tri-methylation of H3K27 (H3K27me2 and H3K27me3) and repress chromatin. The PRC1 complex binds to chromatin bearing the H3K27me3 mark. PRC1 is composed of ubiquitin ligases RING1A and RING1B together with BMI1, MEL18 (PCGF2), and NSPC1 (PCGF1), with RING proteins functioning to monoubiquitinate histone H2A at lysine 119 (H2AK119ub1) (108). Both PRC1 and PRC2 are required to maintain gene suppression, and remain associated with condensed chromatin, even in the absence of the initial trigger. Although PRC1 usually follows the activity of PRC2, there are some reports where PRC2 silenced genes do not contain the PRC1-mediated H2AK119ub1 mark (108).

The components of PRC2 are upregulated in many malignancies such as melanoma, lymphoma, and breast cancers. High expression of EZH2 is seen in ovarian cancer, correlating with advanced stage and is a predictor of poor survival (109). Furthermore, higher levels of EZH2 have been seen in a subpopulation of ovarian cancer cells with stem cell-like properties at relapse following platinum-based chemotherapy. Down-regulation of EZH2 in these stem cell-like populations in ovarian cancer cell models reduced anchorage-independent growth and tumor growth *in vivo* (110). Furthermore, down-regulation of EZH2 was shown to resensitize cisplatin resistant ovarian cancer cells to cisplatin and decrease H3K27me3 levels (111). In line with this discovery, down-regulation of EZH2 leads to re-expression of p21^waf1/cip1^, subsequently promoting apoptosis (112).

The PRC2 complex proteins rely on association with molecules that have DNA-binding abilities such as the lncRNAs *HOTAIR* and *Xist* or the transcription factor JARID2 to direct it to its target (108). A number of repressed or deleted miRNAs associated with ovarian cancer, including miR-199a, miR-214, and miR-26a (89, 90), are also predicted (miRTarBase)^1^ and/or reported to directly target EZH2 suggesting a possible mechanism of its over-expression (113, 114). Interestingly, the tumor suppressor BRCA1 negatively modulates PRC2 by interacting with EZH2 due to overlap in the BRCA1-binding region and *HOTAIR* binding domain of EZH2 (115). *HOTAIR* reprograms luminal breast cancer cells into an aggressive, basal-like state in the absence of functionally wild-type BRCA1 (115). Although the dominant mechanism of PRC2 function is *via* the H3K27me3 repressive mark on target loci, multiple epigenetic mechanisms could also be involved in PRC2-mediated gene silencing since EZH2 and EED are reported to interact with DNMTs and HDACs (25). Unlike EZH1, which is present in both dividing and differentiated cells, EZH2 expression is specific to actively dividing cells (108), making it an attractive therapeutic target for cancer.

## Rise of Epigenetic Therapies Targeting Histone Modifications

**Figure 3:**
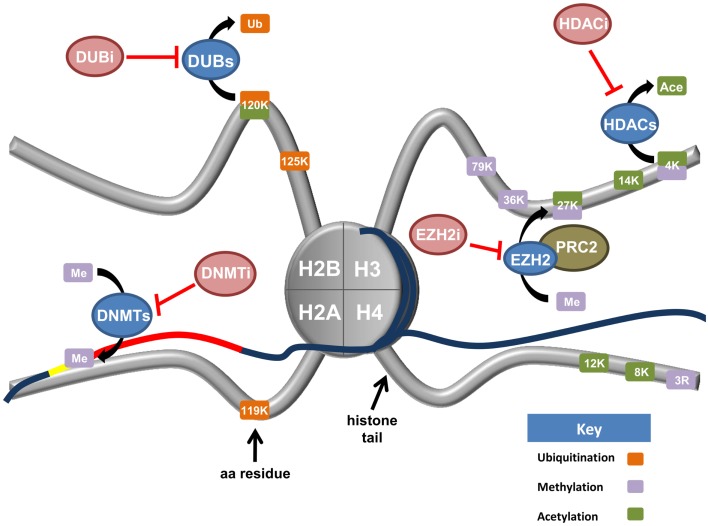
**Current and upcoming therapies for the targeting of epigenetic modifiers in ovarian cancer**. Tumor suppressor genes are commonly silenced in ovarian cancer through epigenetic writers and erasers (blue ovals). These proteins regulate a variety of modifications including DNA methylation (DNMTs), histone methylation (EZH2), the removal of both histone acetylation (HDACs), and histone monoubiquitination (DUBs). Various inhibiting agents (red ovals) have been designed to stop the action of these enzymes. DNA methyltransferases (DNMTs) silence tumor suppressor genes (red line) by hypermethylation of CpG islands in gene promoters (yellow line). Consequently, DNMT inhibitors (DNMTi) are currently being trialed in ovarian cancer cell models with value in the reactivation of a tumor suppressive phenotype. Deubiquitinating enzymes (DUBs) function to cleave ubiquitin from their target proteins. Recent research has demonstrated H2Bub1 is lost in ovarian cancer, implicating H2Bub1-specific DUBs. H2Bub1-associated DUB inhibitors (DUBi) may be viable treatments for ovarian cancer. The histone methyltransferase EZH2 is a member of the Polycomb repressive complex 2 (PRC2). EZH2 functions to tri-methylate lysine 27 of histone H3 (H3K27), a repressive chromatin mark. Consequently, EZH2-inhibitors (EZH2i) are currently being trialed to remove this repressive mark. Histone deacetylase (HDACs) remove acetyl groups from specific histone residues. HDAC inhibitors (HDACi) prevent this enzymatic function, facilitating gene transcription.

Knowledge of epigenetic modifications and the enzymes that regulate them underpin new options for the treatment of ovarian cancer. Drugs specifically targeting DNA methylation will not be discussed but we recommend a recent review addressing this topic (116). In this section, we will examine the rapidly expanding field of drugs targeting histone modifications and the enzymatic machinery driving these changes with the view of application for the treatment of ovarian cancer. Figure 3 depicts histone modifying enzymes currently being targeted or in pre-clinical models for ovarian cancer.

### Histone deacetylase inhibitors

Histone deacetyltransferase inhibitors have been demonstrated to decrease cancer cell growth, induce apoptosis and promote cell differentiation (117). Currently, there exists a wide variety of compounds that can function as HDAC inhibitors including; organic hydroxamic acids, short-chain fatty acids, benzamides, cyclic tetrapeptides, and sulfonamides (118). Many different type of agents derived from these fundamental families are currently going through clinical trials. Of the current HDAC inhibitors, three have been tested in ovarian cancer; suberoylanilide hydroxamic acid (SAHA), valproic acid (VPA), and Romidepsin, either as standalone treatments or in conjunction with DNA damaging agents such as cisplatin. Although this section will focus on FDA approved HDAC inhibitors, other HDAC inhibitors have also recently shown for the potential treatment of ovarian cancer. The HDAC inhibitor M344, which is specific for HDAC6 and to a lesser extent HDAC1, has been shown to promote growth inhibition, cell cycle arrest, and apoptosis, as well as inhibit BRCA1 expression in ovarian cancer cell lines (119, 120). The HDAC inhibitor Trichostatin A (TSA), which specifically inhibits class I and II mammalian HDAC families, has been shown to increase p73 expression and promote Bax-dependent apoptosis in cisplatin resistant ovarian cancer cells (121).

#### SAHA (Vorinostat®)

Suberoylanilide hydroxamic acid has showed promising results in a number of *in vitro* models of ovarian cancer. Early research demonstrated that SAHA was capable of promoting cell cycle arrest, apoptosis, and caspase 3 activation, as well as decrease cell viability in ovarian cancer cell lines and isolated primary cancer cells (122–124). More recent studies have demonstrated that SAHA works effectively in conjunction with paclitaxel in ovarian cancer cell lines (125), while a SAHA–decitabine combination inhibited ovarian cancer cell growth in both *in vitro* and in xenograft models, in addition to increasing the expression of imprinted tumor suppressor genes, increasing apoptosis, cell cycle arrest, and autophagy (126). Further work has shown SAHA to be effective in combination with cisplatin in platinum resistant ovarian cancer cells (127, 128); however, the mechanism of this effectiveness is still poorly defined. Research has linked SAHA treatment to growth arrest, apoptosis and differentiation in a wide range of cancers. The anti-proliferative effect of SAHA has been suggested to be a result of the accumulation of acetylated proteins, including; BCL6, p53, Hsp90, and the core histones (129). Currently, Vorinostat has been through phase I and II clinical trials for ovarian cancer. Phase II trials in women with recurrent platinum-refractory ovarian cancers showed little benefit when this drug was used as a single agent, although the drug was well tolerated (130). Vorinostat was FDA approved in 2006 for the treatment of Cutaneous T-cell lymphoma (CTCL).

#### Valproic acid (Valproate®)

Another HDAC inhibitor showing promise is the short-chain fatty acid drug VPA. Valproate has the advantage of already being a well establish FDA approved drug, used clinically as an anticonvulsant. VPA acts to directly inhibit HDAC activity; however, the specific details of how it exerts its effects are still unclear. Early research demonstrated that VPA promoted cell cycle arrest and apoptosis in ovarian cancer cell lines (122). VPA was also demonstrated to sensitize ovarian cancer cell lines to cisplatin treatment and to resensitize cisplatin resistant cells to treatment (131). More recent research showed that VPA was effective at treating a mouse subcutaneous xenograft model of ovarian cancer, as well as ovarian cancer cell lines (132). This same study demonstrated that VPA treatment resulted in an increase in E-cadherin expression, while decreasing MMP9 and vascular endothelial growth factor (VEGF). Similar to SAHA, VPA has been shown to act as an effective treatment against ovarian cancer cells by itself and in combination with other drugs. Combination treatments of ovarian cancer cell line models with VPA and the Aurora Kinase inhibitor VE465 showed increased apoptosis compared to VE465 alone (133). Monti and colleagues offer an extensive review of VPA mechanisms (134).

#### Romidepsin (FK228, Istodax®)

Romidepsin is a class I HDAC inhibitor, which received FDA approval in 2009 for the treatment of CTCL. In a biological system Romidepsin functions as a pro-drug whereby its reduction results in the release of a thiol, which blocks the activity of Zn-dependent histone deacetylase through its interactions with the zinc atom present in the deacetylase’s binding domain (135). A study has demonstrated that Romidepsin inhibited cell viability and induced apoptosis in ovarian cancer cell lines (136). More recent work by the same group demonstrated that Romidepsin worked effectively in combination with cisplatin increasing cell apoptosis in *in vitro* and *in vivo* models (137). Romidepsin is currently in phase 2 trials for ovarian cancer^2^ (NCT00085527).

### Deubiquitinases – targeting monoubiquitinated histone H2B (H2Bub1)

Ubiquitin is traditionally thought of in the context of polyubiquitination that leads to protein degradation *via* the ubiquitin–proteasome system; however, monoubiquitination of histones H2A and H2B are clear instances of alternative roles for ubiquitin in transcription and DNA repair (45, 46, 138). DUBs are proteases that cleave ubiquitin from target proteins, including core histone proteins, and are recognized as important regulators of the ubiquitin–proteasome system. Given that DUBs occur upstream of the proteasome, they have the potential to show greater specificity and less toxicity compared to FDA approved proteasome inhibitors such as Velcade^®^ (bortezomib) or Kyprolis^®^ (carfilzomib). For these reasons, extensive efforts are currently being focused on DUBs as drug targets (139, 140). Currently, no specific DUB inhibitor has entered clinical trials; however, DUB inhibitors have shown promise in pre-clinical models, including P0591, an inhibitor of USP7, that has amongst its substrates H2Bub1 and the p53 regulator HDM2 (141). In studies of multiple myeloma, P5091 was demonstrated to induce apoptosis in both bortezomib refractory multiple myeloma cells and animal tumor models (141). Further, there is considerable interest in the H2Bub1-targeting DUB USP22 given its membership of an 11-gene panel termed the “*Death-from-Cancer*” signature that predicts rapid disease recurrence, distal metastasis, and poor response to therapy (142).

### Histone methyltransferases – targeting EZH2

Like DNMTs, HMTases such as EZH2 can be targeted for therapy. 3-Deazaneplanocin A (DZNep) was the first indirect inhibitor of EZH2, leading to decreased global levels of H3K27me3 and restoration of expression of genes involved in growth inhibition or apoptosis (143). It was subsequently discovered that DZNep also inhibited other HMTases (60, 143, 144). Specific inhibitors of EZH2 have since been developed including GSK126, EPZ005687, and EI1 (60, 145–147). Recently, a peptide-based inhibitor, SAH-EZH2, was developed to target the PRC2 by disrupting EZH2/EED interactions (148).

Results of preliminary studies of EZH2-inhibitors in combination with other drugs have been encouraging. DZNep was shown to enhance the anti-proliferative effects of Gemcitabine in pancreatic cancer cells (149). The combination of both DNA demethylating agents (5-aza-2′-deoxycytidine) and DZNep targeting histone methylation has shown promise in cell line models of leukemia (150). A report on the use of DZNep treatment of the ovarian cancer cell line A2780 showed reduction in proliferation, an increase in apoptosis, inhibition of migration, and upregulation of E-cadherin expression (151). It remains to be seen whether these therapeutic strategies may be of value for the treatment of ovarian cancer.

## Concluding Remarks

Targeting of histone modifications and the enzymes regulating them in the ovarian cancer epigenome represents, to date essentially an unmet opportunity. With further focus on this field, it is probable that within the next decade new drugs targeting HDACs, HTMases, and DUBs will emerge as the next generation of cancer therapeutics. Whether these drugs will be most efficacious as single agents, or in combinatorial approaches with more traditional DNA damage-based chemotherapeutics, or with other perhaps yet to be developed molecular based targeted drugs remains to be determined. It is clear that complex networks of histone cross-talk will need to be understood and markers of histone dysregulation will need to be identified to ensure that patients receive maximal benefit from these therapies (152). In summary, the field of epigenomic-based histone therapies promises to offer a new generation of cancer therapeutics giving fresh hope for the treatment of women with ovarian cancer.

## Author Contributions

Deborah J. Marsh., Jaynish S. Shah, and Alexander J. Cole conceived, wrote, and critically revised this manuscript.

## Conflict of Interest Statement

The authors declare that the research was conducted in the absence of any commercial or financial relationships that could be construed as a potential conflict of interest.

## Footnotes

^1^http://mirtarbase.mbc.nctu.edu.tw/

^2^http://clinicaltrials.gov/ct2/crawl/42
